# Chemistry of Homocysteine Thiolactone in A Prebiotic Perspective

**DOI:** 10.3390/life9020040

**Published:** 2019-05-16

**Authors:** Ibrahim Shalayel, Yannick Vallée

**Affiliations:** CNRS, Département de Chimie Moléculaire, Univ. Grenoble Alpes, Campus, F-38058 Grenoble, France; ibrahim.shalayel@univ-grenoble-alpes.fr

**Keywords:** origin of life, prebiotic chemistry, thioester world, amino acids, homocysteine

## Abstract

Homocysteine is a non-proteinogenic sulfur-containing amino acid. Like cysteine, it can form disulfide bridges and complex metallic cations. It is also closely related to methionine, the first amino acid in the synthesis of all contemporary proteins. Furthermore, its cyclized form, a five-membered ring thiolactone, is stable in acidic and neutral water. Here, we demonstrate that this thiolactone may have been formed in the primitive ocean directly from the Strecker precursor of homocysteine, an aminonitrile. Even though it is poorly reactive, this thiolactone may be open by some amines, yielding amides which, in turn, could be the precursors of longer peptides.

## 1. Introduction

Two sulfur amino acids are on the list of the 20 classic proteinogenic amino acids. Thanks to their ability to form disulfide bridges, cysteine residues are involved in protein folding. They are also of the highest importance in many catalytic sites [1], especially catalytic triads [2], and are often linked to metal cations, for example in zinc fingers [3] and in iron-sulfur clusters [4]. A methionine residue is introduced at the beginning of all proteins in Archaea and Eukaryotes. *N*-Formyl-methionine is used in prokaryotes. Indeed, this rule suffers no exception, whereas it is known that the start codon, usually AUG, which also codes for methionine within proteins, is sometimes replaced by alternative codons [5]. At the beginning of the synthesis of a protein a methionine residue is thus introduced, even if the initiation codon should code for another residue.

**Figure 1:**
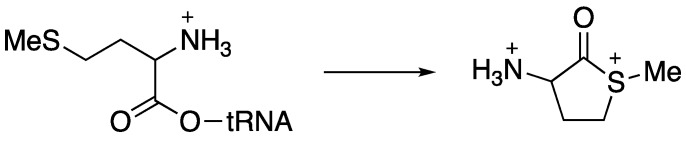
Formation of a cyclic sulfonium from methionine.

We have proposed that this could be explained by the fact that, in the prebiotic world, methionine has been “chosen” before the start codon [6]. In other words, the choice of methionine would have predated the development of any genetic information. This would imply that methionine has a property that clearly distinguishes it among all the amino acids. This property should facilitate its activation, so that it would form a dipeptide much faster than any other amino acid. Jakubowski proposed that a cyclic derivative of methionine, a five-membered ring acyl sulfonium, was formed during in vitro tRNA aminoacylation catalyzed by *Escherichia coli* methionyl-tRNA synthase (Figure 1) [7]. Such an intermediate would be highly electrophilic and if it could be formed directly from methionine (without the need to use a complex catalyst), it would allow very effective access to dipeptides under prebiotic conditions. However, we have never been able to form it in water from methionine itself or from any of its simple derivatives [8].

It is tempting to imagine that homocysteine, in which the methylthio group of methionine is replaced by the much more reactive thiol function, could have played some role as a more reactive substitute for methionine, but also as an alternative to cysteine. Even though homocysteine is not a proteinogenic amino acid, and indeed exhibits a proven [9] toxicity, it is present in living cells where, among other things, it participates in the metabolisms of cysteine and SAM [10].

Recently we have reported the synthesis of various dipeptides starting from cysteine and aminonitriles under plausible prebiotic conditions [11]. Such dipeptides, as well as longer peptides containing thiol groups, could have been promoters of other reactions in the “thioester world” proposed by De Duve [12,13], perhaps in a way similar to what is happening today in the non-ribosomal synthesis of peptides [14]. Indeed, the participation of homocysteine [15] and homocysteine thiolactone [12] in primitive peptide synthesis has already been proposed.

HCN was probably present on the primitive earth [16,17]. It is thus plausible that homocysteine, as other amino acids, was synthesized in the primitive ocean via a Strecker reaction [18]. If so, as already proposed (but not tested) by Van Trump and Miller [19], the starting aldehyde should have been 3-mercaptopropanal [20], itself obtained thanks to the [1,4] addition of H_2_S onto acrolein. If this was true, then nitrile **1** was an intermediate in the synthesis of homocysteine. We decided to synthesize and to study the behavior of this nitrile.

## 2. Experimental Section

For experiments mimicking prebiotic conditions, products were identified in reaction mixtures. No attempt at purifying them was made.

NMR monitored reactions were run in D_2_O solutions, in NMR tubes. NMR apparatus: Bruker Avance III 400 or 500. Copies of spectra are available in Supplementary Materials.

For mass experiments, H_2_O was used as the solvent. High-resolution mass spectra were recorded on a Waters G2-S Q-TOF mass spectrometer or on an LTQ Orbitrap XL (Thermo Scientific, Waltham, MA, USA) spectrometer. Low-resolution ESI analysis was performed on an Amazon speed (Brucker Daltonics, Bremen, Germany) IonTrap spectrometer.

**Scheme 1:**
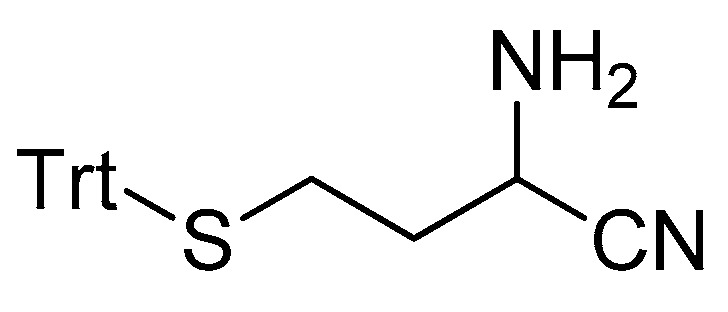
2-amino-4-(tritylthio)butanenitrile 3.

Aldehyde **2** (145 mg, 0.43 mmol) was dissolved in 5 mL THF. Trimethylsilyl cyanide (55.5 mg, 0.56 mmol) and then ZnI_2_ (25.5 mg, 0.08 mmol) were added. After stirring for 15 min at room temperature, 1 mL of ammonia/MeOH solution was added to the mixture and left stirring for 2 h. Then, it was extracted three times with ethyl acetate. The organic layer was dried over Na_2_SO_4_ and evaporated under reduced pressure. The crude product was purified by silica gel chromatography (95–70% pentane/ethyl acetate) to give nitrile **3** (Scheme 1) in 87% yield.

**^1^H-NMR (500 MHz, CDCl_3_) (δ, ppm)**: 7.34 (dd, *J* = 8.4, 1.9 Hz, 6H), 7.21 (t, *J* = 7.7 Hz, 6H), 7.16–7.12 (m, 3H), 3.55 (t, *J* = 7.2 Hz, 1H), 2.31 (td, *J* = 7.2, 1.5 Hz, 2H), 1.58–1.47 (m, 2H), 1.33 (s, 2H); **^13^C-NMR (125 MHz, CDCl_3_) (δ, ppm)**: 144.55, 129.58, 128.05, 126.86, 121.59, 67.07, 42.23, 34.52, 27.69. **HRMS** (ESI): calcd for C_23_H_22_N_2_SNa [M + Na]^+^: 381.1396, found: 381.1390.

**Scheme 2:**
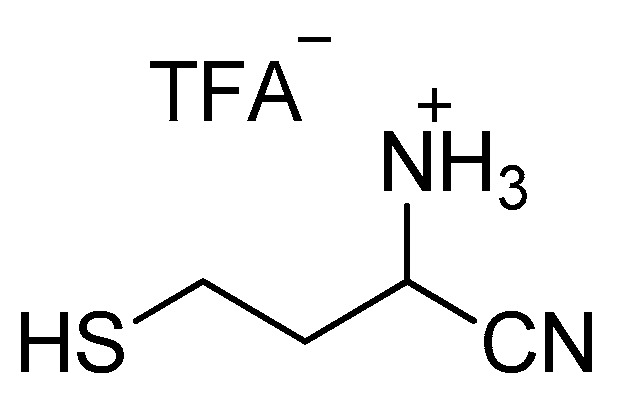
1-cyano-3-sulfanylpropan-1-aminium trifluoroacetate 1.CF_3_CO_2_H.

Aminonitrile **3** (30 mg, 0.083 mmol) was dissolved in dichloromethane (2 mL). Triisopropylsilyl hydride (69 µL, 0.33 mmol) and trifluoroacetic acid (64 µL, 0.83 mmol) were added. The mixture was stirred for 30 min at room temperature and then was extracted with D_2_O (1 mL). Finally, the aqueous layer was left under vacuum at room temperature to remove the remaining dichloromethane to provide a D_2_O solution of product **1** (Scheme 2) as its trifluoroacetate.

**^1^H-NMR (500 MHz, D_2_O) (δ, ppm)**: 4.49 (dd, *J* = 9.2, 5.8 Hz, 1H), 2.60-2.55 (m, 1H), 2.48-2.42 (m, 1H), 2.15-2.08 (m, 1H), 2.04-1.96 (m, 1H); **^13^C-NMR (126 MHz, D_2_O) (δ, ppm)**: 115.29, 40.18, 33.85, 19.07.

**Scheme 3:**
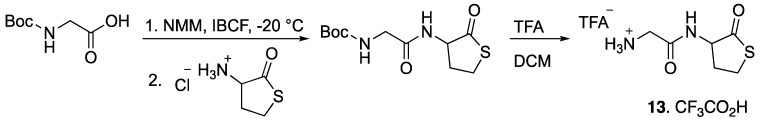
Synthesis of an authentic sample of compound 13.

**Scheme 4:**
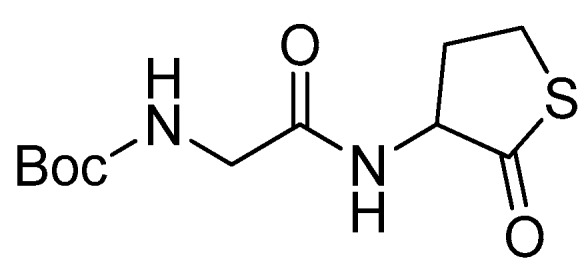
Tert-butyl (2-oxo-2-((2-oxotetrahydrothiophen-3-yl)amino)ethyl)carbamate.

Boc-Gly-OH (500 mg, 2.85 mmol) was dissolved in 30 mL THF. N-methyl morpholine (0.85 mL, 7.7 mmol) and isobutyl chloroformate (0.55 mL, 4.28 mmol) were added at -20 °C (Scheme 3). After 15 min, Hcy-thiolactone hydrochloride (526 mg, 3.48 mmol) was added to the mixture which was then stirred overnight at room temperature. The mixture was concentrated under reduced pressure, then water was added, and the organic layer was extracted three times with ethyl acetate. The organic phase was evaporated under reduced pressure. The crude product was purified over a silica column with 90–50% pentane/ethyl acetate to provide the desired product (Scheme 4) as a white solid (88%).

**^1^H-NMR (500 MHz, CDCl3) (δ, ppm)**: 7.11 (s, 1H), 5.59 (s, 1H), 4.62 (d, *J* = 5.5 Hz, 1H), 3.65–4.02 (m, 2H), 3.34–3.40 (m, 2H), 2.78 (s, 1H), 1.94–2.16 (m, 1H), 1.44 (s, 9H); **^13^C-NMR (125 MHz, CDCl_3_) (δ, ppm):** 205.39, 170.42, 156.18, 80.20, 59.03, 44.12, 31.26, 28.30, 27.43; **HRMS** (ESI): calcd for C_11_H_18_N_2_O_4_SNa [M + Na] ^+^: 297.0885, found 297.0890.

**Scheme 5:**
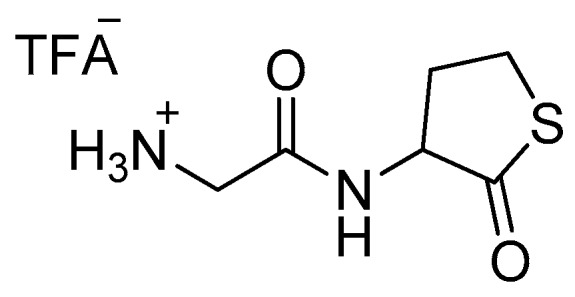
2-oxo-2-((2-oxotetrahydrothiophen-3-yl)amino)ethanaminium trifluoroacetate 13.CF_3_CO_2_H.

The previously obtained Boc-derivative (80 mg, 0.29 mmol) was dissolved in dichloromethane (2 mL). Triisopropylsilyl hydride (0.5 mL) and trifluoroacetic acid (0.5 mL) were added to this mixture which was then left stirring for 30 min at room temperature. The solution was evaporated, and the crude product was washed three times with dichloromethane. The product was then evaporated under reduced pressure to afford **13** (Scheme 5) as its trifluoroacetate (quantitative).

**^1^H-NMR (500 MHz, D_2_O) (δ, ppm)**: 4.67, (q, *J* = 6.7 Hz, 1H), 3.79 (d, *J* = 3.0 Hz, 2H), 3.27– 3.42 (m, 2H), 2.55–2.6 (m, 1H), 2.11–2.20 (m, 1H); ^13^**C-NMR (125 MHz, D_2_O)**: 210.31, 167.22, 59.33, 40.38, 29.79, 27.47. **HRMS** (ESI): Calcd for C_6_H_11_N_2_O_2_S [M + H] ^+^: 175.0536, found 175.0535.

## 3. Results

**Figure 2:**
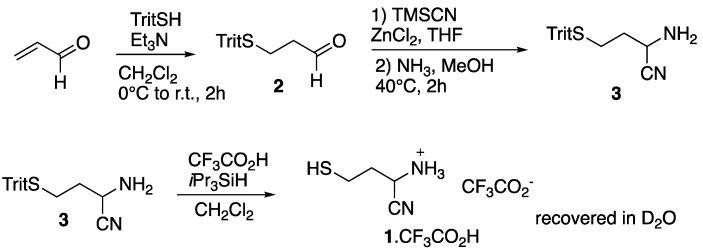
Synthesis of the aminonitrile 1 from acrolein.

**Figure 3:**
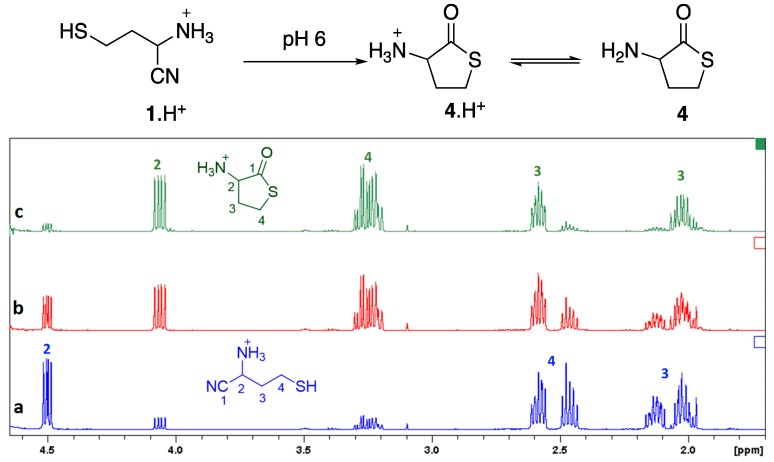
^1^H NMR of aminonitrile **1** in D_2_O (pH 1) immediately after its synthesis (spectrum **a**), and of its evolution product (pH 6), thiolactone **4** (**b**, after 1 h; **c**, after 2h). Concentration 0.083 mol/L, 24 °C. The signals for protons on carbons 2, 3 and 4 for both compounds are identified.

As 3-mercaptopropanal itself is a poorly stable compound [20], we decided to synthesize the aminonitrile **1** from the protected thiol **2** (Figure 2). Acrolein was reacted with triphenylmethanethiol and the obtained aldehyde **2 [21]** was treated with trimethylsilyl cyanide and ammonia [22] to give the aminonitrile **3**. The S-trityl bond was then cleaved by treatment with trifluoroacetic acid in dichloromethane, after what the trifluoroacetate salt of **1** was recovered in D_2_O and analyzed by NMR spectroscopy (Figure 3). At the initial acidic pH (*ca.* 1) the observed spectrum agreed with proposed structure **1**. The proton α to the nitrile group appeared as a dd at ca. 4.49 ppm. The two methylene groups were seen as multiplets centered at ca. 2.51 (CH_2_S) and 2.06 ppm. These signals were accompanied by smaller signals, including a dd at 4.06 ppm. The pH was then rapidly fixed at six by addition of some sodium bicarbonate. Under these plausible prebiotic conditions, the dd at 4.06 ppm still grew. The final spectrum was consistent with the formation of known (commercially available) homocysteine thiolactone **4**.

**Figure 4:**
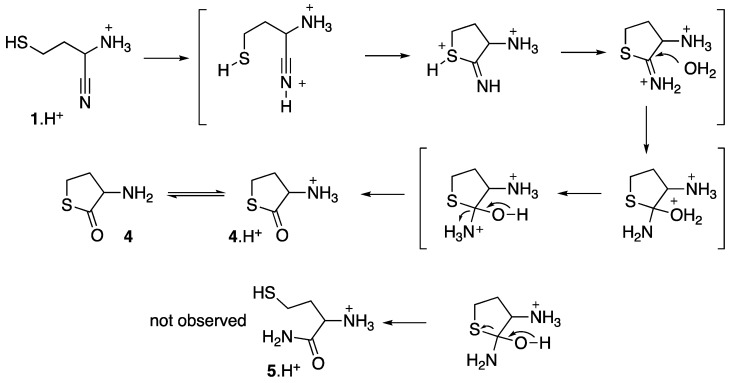
Proposed mechanism for the cyclization of **1** into **4**.

A proposed mechanism for this cyclization is presented in Figure 4. It is shown using the ammonium forms of all the intermediates. It might be, however, that free amine forms intervene. It is also possible that the thiol group could be deprotonated. However, the used conditions are probably not basic enough to allow such deprotonation, so that a classical Pinner [23] type mechanism seems more plausible. As it is usual [24] that the hydrolysis of thioimidates yields mixtures of thioesters and amides, it is noticeable that in this case, the amide **5**, which would result from an alternative final ring opening, was not observed.

We found thiolactone **4** to be stable in D_2_O at pHs up to seven. At higher pHs it started to hydrolyze slowly to give homocysteine. However, as it is probable that the primitive ocean’s pH was slightly acidic [25], we believe that the final product of the Strecker reaction of 3-mercaptopropanal in this ocean would be **4**. Indeed, as it has been proposed that non-cyclic thioesters were not stable on the early Earth [26], it is possible that **4** was the only amino thioester able to accumulate in the primitive ocean.

**Figure 5:**
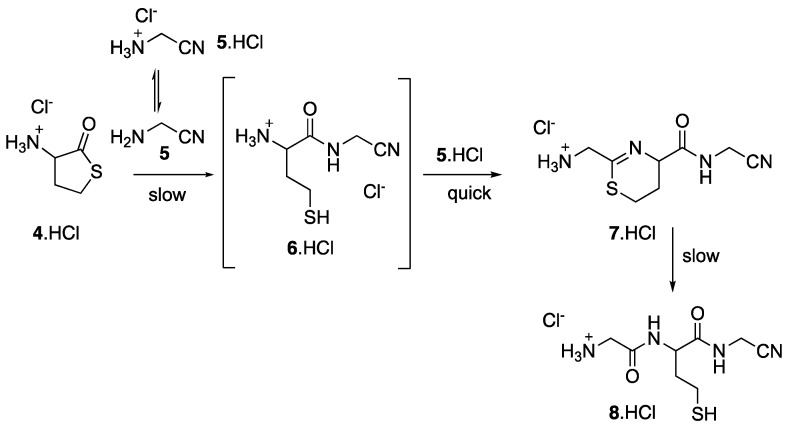
Reaction of thiolactone **4** with aminonitrile **5**.

Even though thiolactone **4** is known to react with amines (such as lysine residues) [9,27], it might be less electrophilic than linear thioesters, as it has been reported for a similar example [28]. However, when we mixed **4** in water (at pH 6) with various amino acids, no amide bond formation was detected. However, **4** reacted with aminoacetonitrile **5** (pH 6, r.t., 2 eq. of **5**), the product of the Strecker reaction of formaldehyde (Figure 5). The first step of this reaction is the opening of the thiolactone ring by the amine function of **6**. Such reaction was undoubtfully favored by the low pKa of **5**, 5.55 [29], to be compared to the pKa, from 9 to 10, of amino acids. Nevertheless, in the conditions we used, this opening step was only slow and never went to completion. Indeed, we did not observe the amidonitrile **6**, because this intermediate reacted with the nitrile function of **5** in a much quicker process giving the dihydrothiazine **7**. We have already reported similar S,N-heterocyclic ring formation [11].

**Figure 6:**
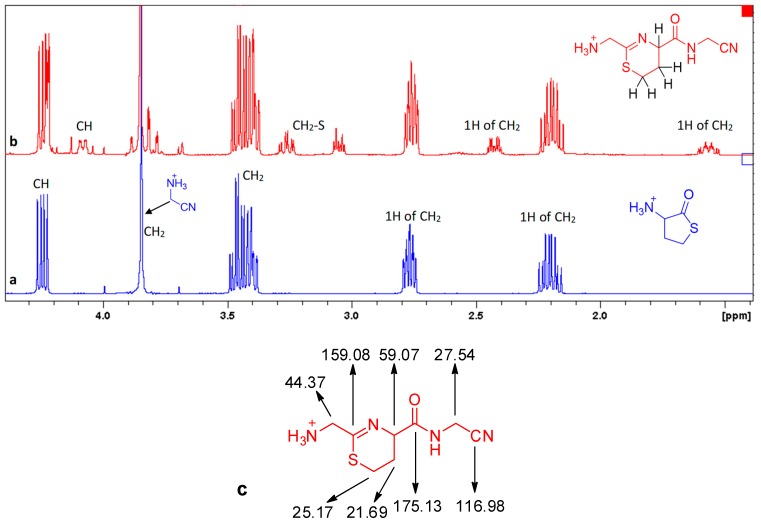
Formation of **7** from **4** (reaction in D_2_O, pH 6, 24 °C, concentration of **4** 0.08 mol/L). **a,** t_0_; **b,** t_0_ + 24 h; **c,** ^13^C NMR data for **7**.

Dihydrothiazine **7** was characterized in the reaction mixture (Figure 6). In the ^1^H NMR spectrums, the most characteristic signals were those of the protons in the dihydrothiazine ring. The CH_2_S group appeared as two doublets of triplets centered at 3.27 and 3.06 ppm. The signals of the two protons of the other CH_2_ were separated by ca. 0.9 ppm, centered at 2.43 and 1.57 ppm. Finally, the proton α to the amide function gave a ddd (*J* = 10, 4 and 2 Hz) at 4.09 ppm. We were also able to attribute all the ^13^C peaks of **7**.

Globally the conversion of **4** into **7** was never higher than 25%. Furthermore, the hydrolysis of **7** into the corresponding nitrile-tripeptide, Gly-Hcy-GlyCN **8,** was also a slow process. The formation of **7** and **8** were confirmed by high resolution mass spectrometry (**7**: Calcd for C_8_H_13_N_4_OS [M + H] ^+^: 213.0810, found 213.0806; **8**: Calcd for C_8_H_15_N_4_O_2_S [M + H] ^+^: 231.0916, found 231.0917).

**Figure 7:**
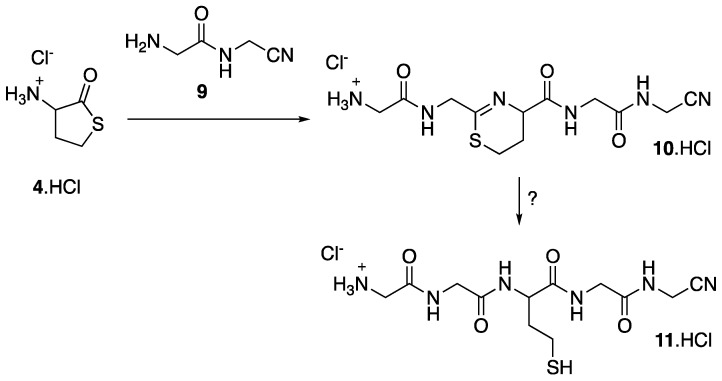
Reaction of thiolactone **4** with amidonitrile **9**.

We also attempted the reaction of **4** with the amidonitrile **9** [11]. In this case, the expected product was the dihydrothiazine **10** (Figure 7). In the ^1^H NMR of the reaction mixture, we observed signals characteristic of the SCH_2_ of the dihydrothiazine ring, and the formation of **10** was confirmed by HRMS (calcd for C_12_H_19_N_6_O_3_S [M + H] ^+^: 327.1239, found 327.1231). However, we were not able to detect the nitrile-pentapeptide Gly-Gly-Hcy-Gly-GlyCN **11**, which should result from the hydrolysis of **10** and the conversion of **4** into **10** remained low (*ca.* 7% estimated from ^1^H NMR of the reaction mixture).

**Figure 8:**
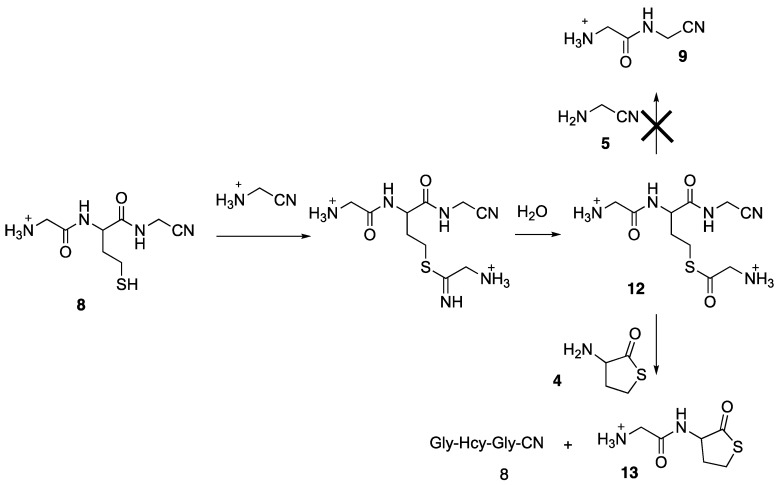
Formation of the aminoacetylated thiolactone **13**.

In a previous paper [11], we have shown that a thioester formed from glycinylcysteine was able to transfer an aminoacetyl group to aminoacetonitrile **5**, hence leading to **9**. We decided to try a similar transfer reaction starting from **8** (Figure 8). This nitrile tripeptide was generated by the method described in Figure 5, but 10 equivalents of **5** were used. We were expecting the formation of **9**, via the thioester **12**, but no **9** was detected. Instead, we noticed the formation of the aminoacetylated thiolactone **13** (21% conversion from **4** determined by ^1^H NMR; ^13^C NMR, 2 C = O at 210.31 and 167.22; see Experimental Section for the synthesis of an authentic sample of this product), meaning that **12** did not react with aminoacetonitrile **5**, but with homocysteine thiolactone **4**. The pKa of **4**, 6.67 [30], is higher than the pKa of **5**, 5.55, so that it is expected that a larger amount of free amine should be present for **5**, compared to **4**. However, it is probable that the very low pKa of **5** also witnesses its low nucleophilicity. This would explain why **4** reacted quicker than **5**.

## 4. Conclusions

Methionine [19] and homocysteine [31] were probably prebiotic molecules. In fact, it might well be that homocysteine was more abundant than cysteine, for which no abiotic synthesis seems obvious (even though Khare and Sagan observed the formation of its disulfide, cystine, under harsh conditions from mixtures of methane, ethane, ammonia, and H_2_S [32]). Furthermore, even if homocysteine was not produced by Strecker reaction, hence not via nitrile **1**, it remains possible that its thiolactone **4** was formed directly from the amino acid. Formation of the five-membered ring is favorable, and this cyclization is known to occur under acidic conditions [33]. As **4** is pretty stable in water up to pH 7, it could have been stored in non-negligible amounts, and used in ring opening reactions by amines and in peptide syntheses [34,35]. If such reaction occurred with a homocysteine thiolactone terminated peptide chain, then it would be quite similar to native chemical ligation [36,37] and would lead to rapid chain extension.

Finally, the truly prebiotic character of the chemistry presented here and in our previous paper [11], largely depends on the presence of large amounts of HCN in the primitive ocean and is based on the assumption that it played a major role [38]. The importance of this chemistry at the origin of life must be weighed against other hypotheses, especially those that are closer to the current metabolism [39,40,41] and compared to other recent developments in the understanding of a possible thioester world [42,43].

## Supplementary Materials

The following are available online at https://www.mdpi.com/2075-1729/9/2/40/s1.
