# Supplementary material for: Chemistry of Homocysteine Thiolactone in A Prebiotic Perspective
Source: Life (Basel). 2019 May 16;9(2):40. doi: 10.3390/life9020040 (PMC6616635; doi:10.3390/life9020040)

**Supplementary material for:**

# Prebiotic chemistry of homocysteine and its thiolactone

Ibrahim Shalayel and Yannick Vallée*

Univ. Grenoble Alpes, CNRS, Département de Chimie Moléculaire, Campus, Grenoble, France

1- Copies of NMR spectra of **3** 2

2- $^{13}C$ NMR of **1** and **4** mixture 4

3- $^{13}C$ NMR of **7** 6

4- $^{1}H$-NMR of ($CH_2$-S) protons of **7** 7

5- Copies of Mass spectrometry of **7** and **8** 8

6- ESI analysis of **4** and **5** reaction mixture 10

<sup></sup>$^{1}$H NMR of **3**

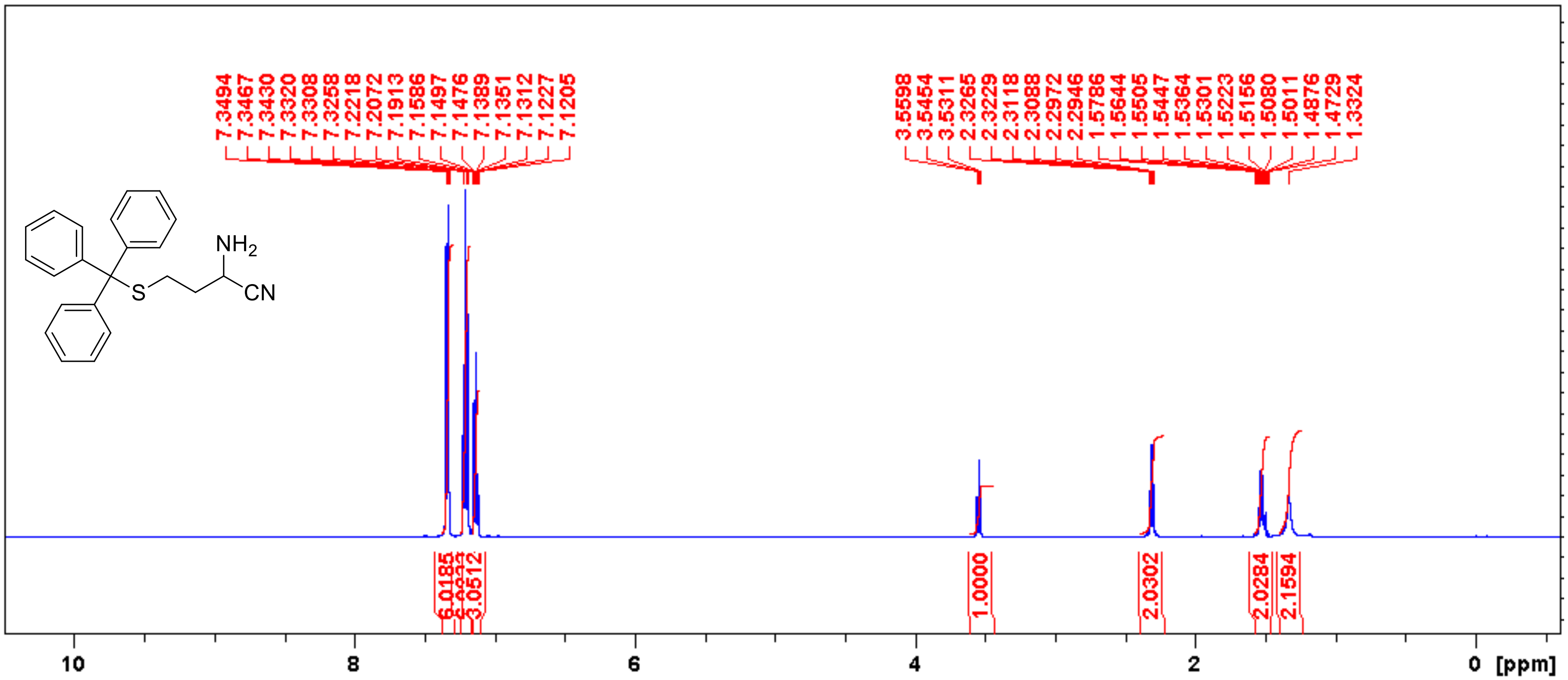

$^{13}C$ NMR of **3**

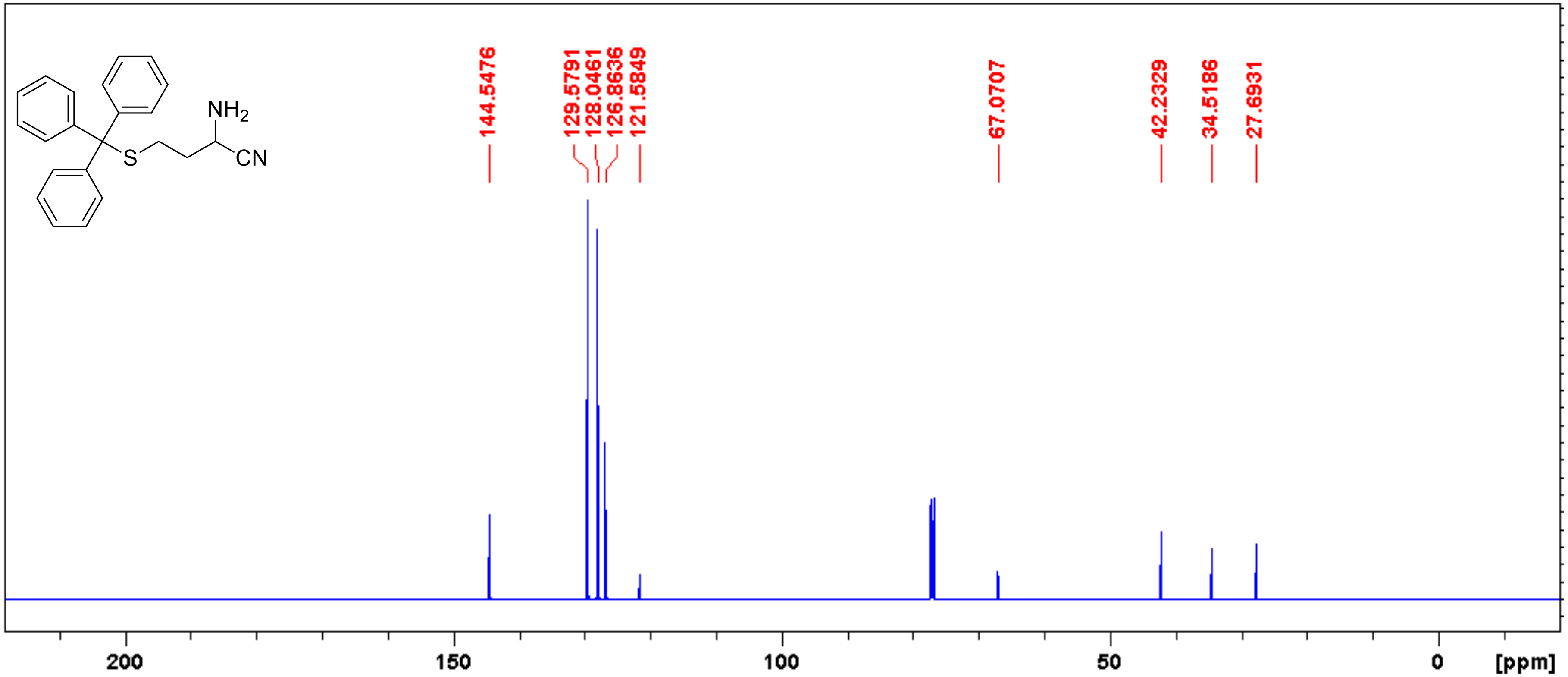
NH2
S
CN
144.5476
129.5791
128.0461
126.8636
121.5849
67.0707
42.2329
34.5186
27.6931
200
150
100
50
0
[ppm]

$^{13}$C NMR of a mixture of **1** and **4**

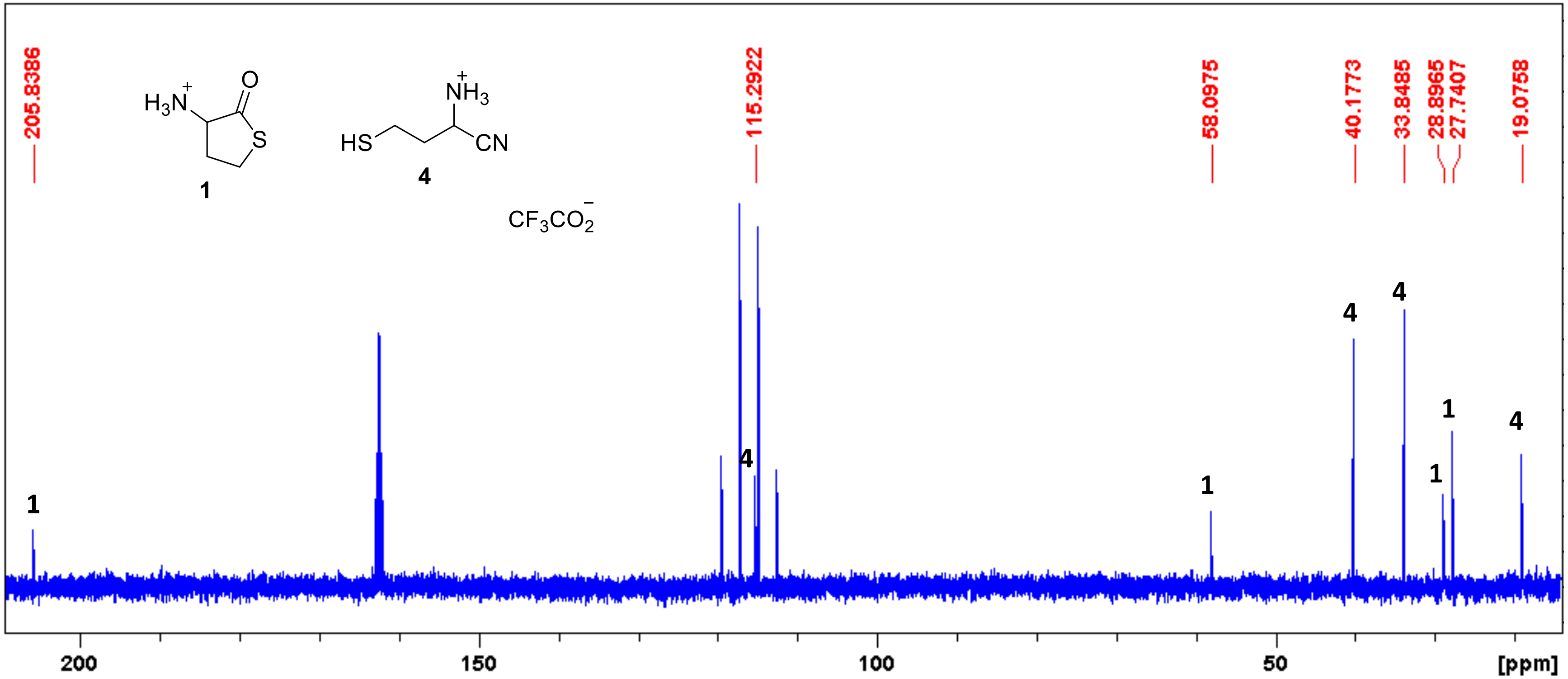

$^{13}$C NMR of Hcy-thiolactone **4**

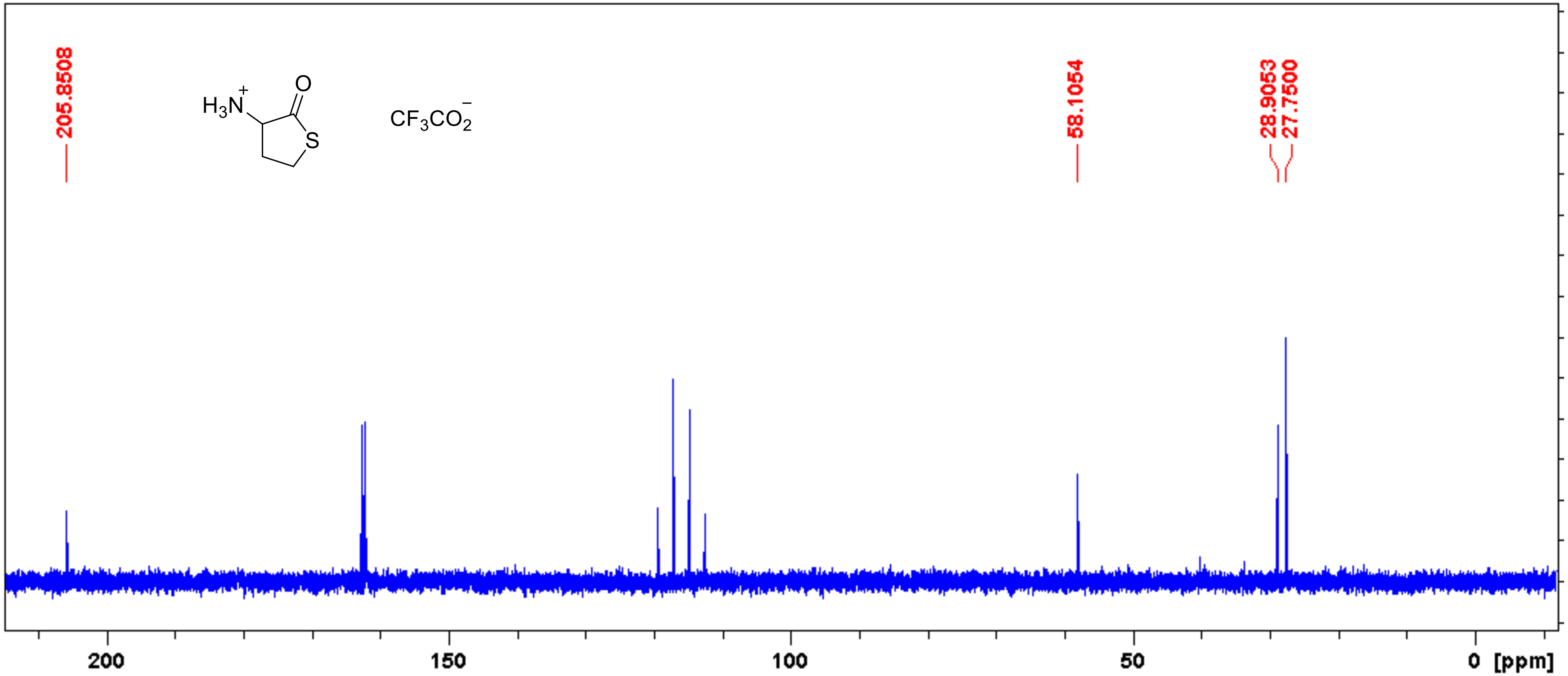

$^{13}$C NMR of a reaction mixture between Hcy-thiolactone **4** and Gly-CN **5**, peaks corresponding to 7 are identified.

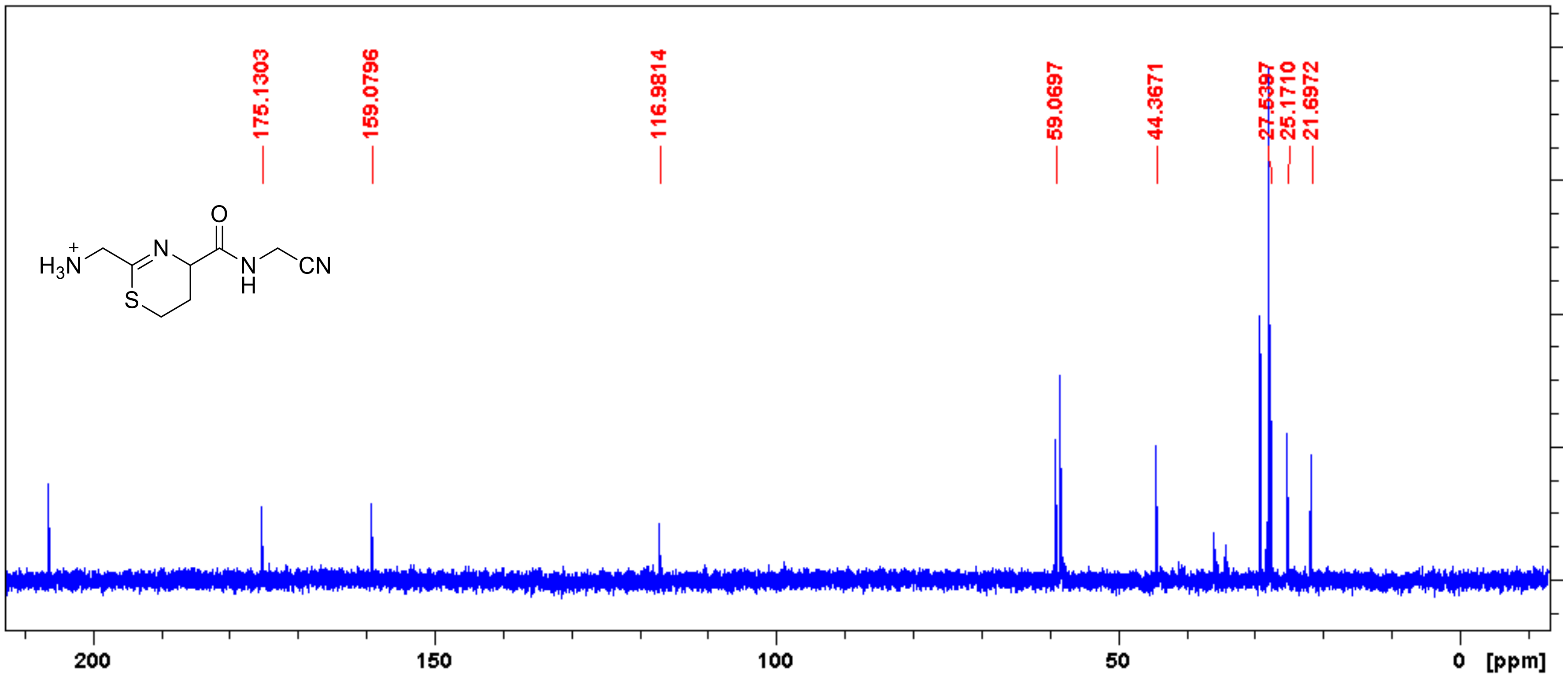

$^{1}$H-NMR of ($CH_2$-S) protons: **a)** dihydrothiazine **7**, **b)** Gly-Hcy dihydrothiazine 2-(ammoniomethyl)-5,6-dihydro-4H-1,3-thiazine-4-carboxylate , **c)** a mixture of **7** and Gly-Hcy dihydrothiazine.

**a)** Gly-Hcy dihydrothiazine cycle (obtained by mixing homocysteine and aminoacetonitrile in $D_2O$ at 45 °C for 2 hours). **b)** Cycle **7** (obtained by mixing Hcy-thiolactone **4** with aminoacetonitrile in $D_2O$ at 45 °C for 3 hours). **c)** Mixture of the two previous solutions together confirming that the observed cycle **7** is not the Gly-Hcy dihydrothiazine cycle.

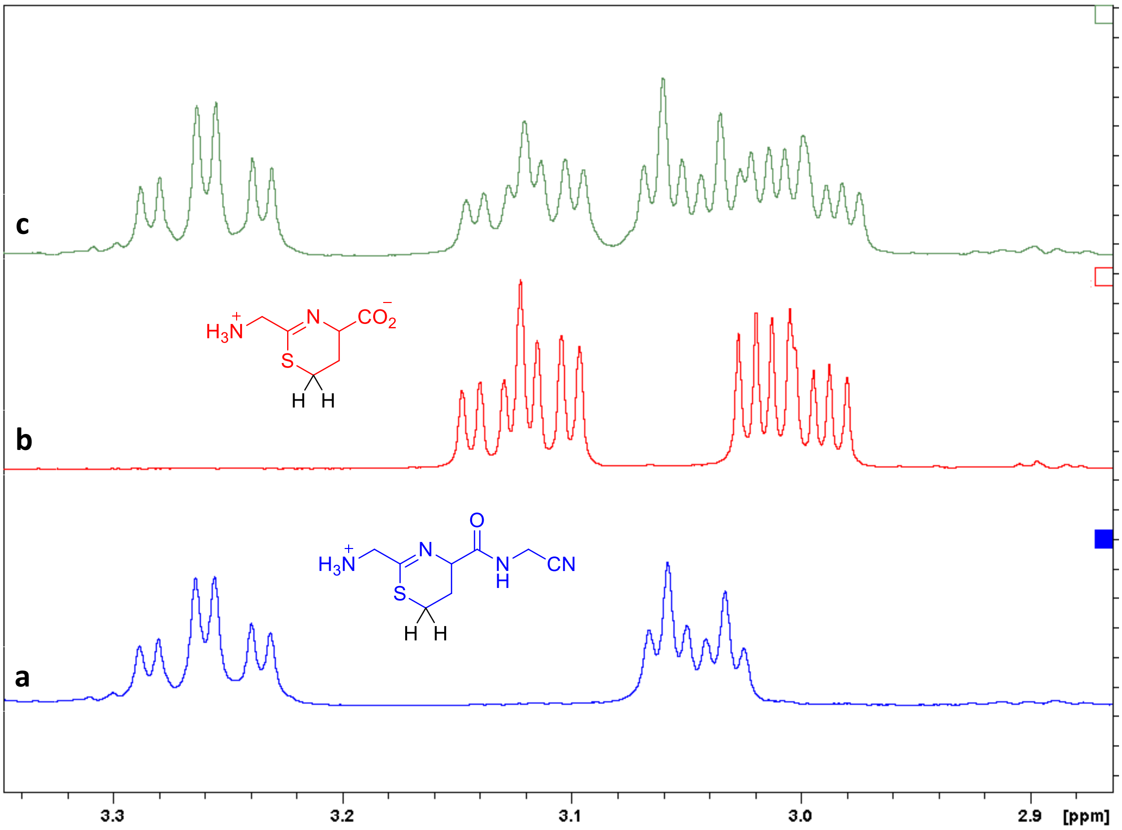

HRMS of **7** in the mixture

calcd for $C_8H_{13}N_4OS$ [M + H] $^+$: 213.0810, found 213.0806

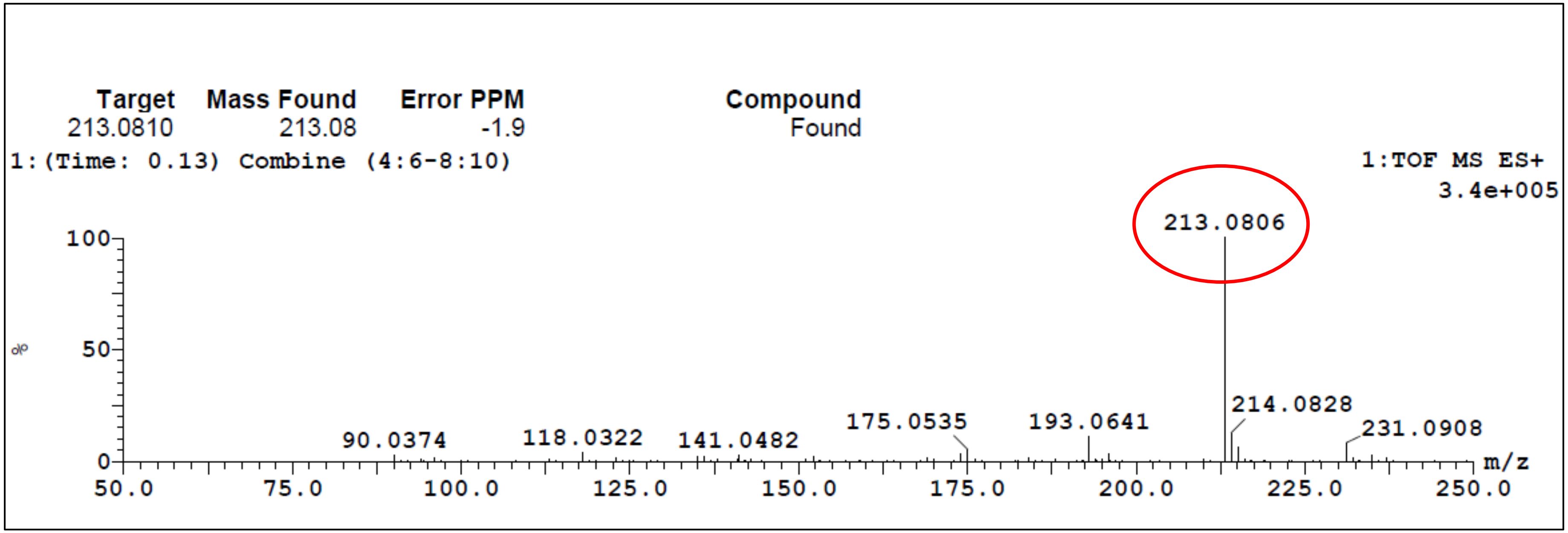

HRMS of **8** in the mixture

calcd for $C_8H_{15}N_4O_2S$ [M + H] $^+$: 231.0916, found 231.0917)

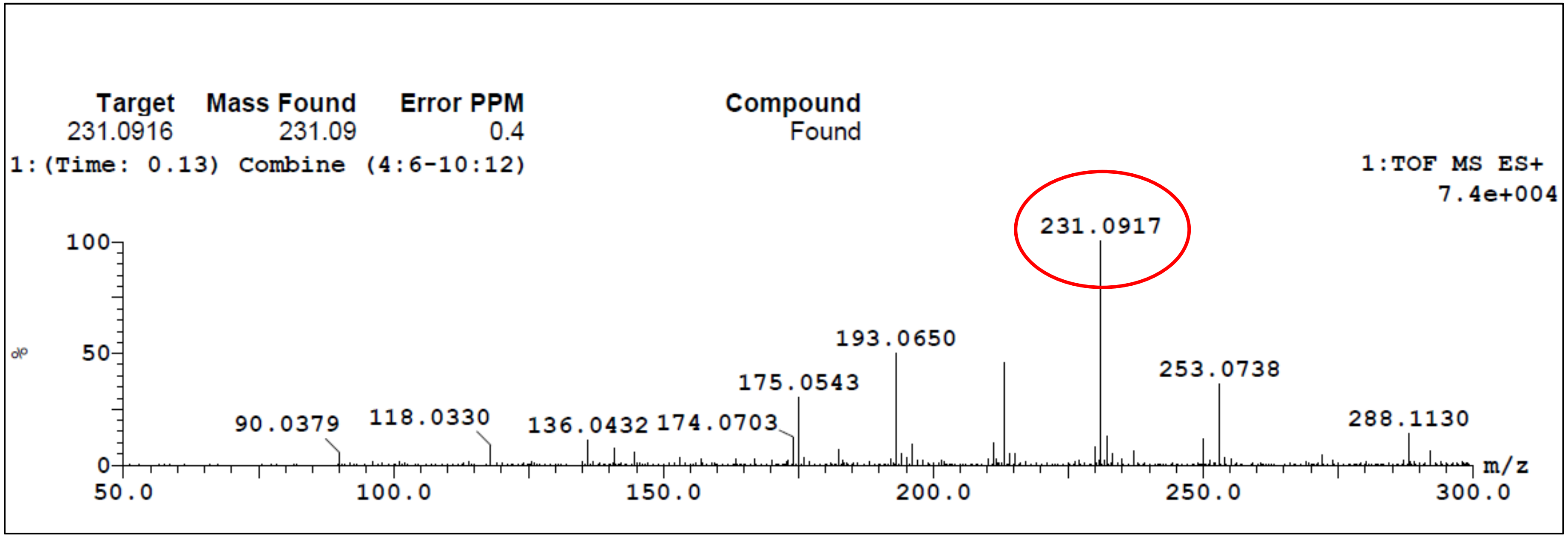

ESI spectrum (law resolution) of **4** and **5** reaction mixture in $H_2O$.

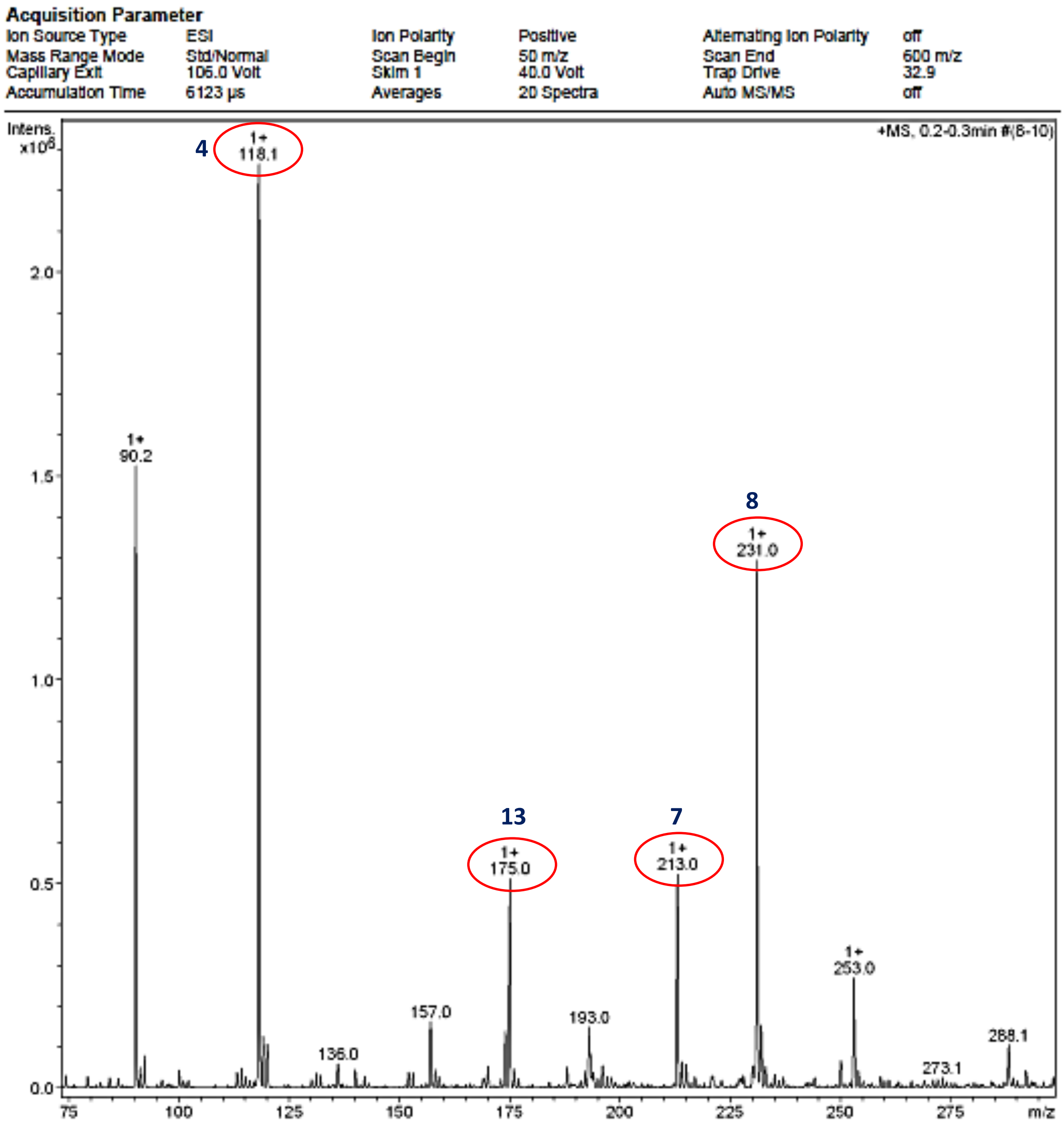

Supplement: Supplementary file 1 [file life-09-00040-s001.pdf]
